# Regional Workshops to Widely Disseminate an Innovative Practice

**DOI:** 10.1093/geroni/igaa057.2744

**Published:** 2020-12-16

**Authors:** Debra Hain, Armiel Suriaga

**Affiliations:** Florida Atlantic University, Boca Raton, Florida, United States

## Abstract

The second stage of the CMS Region IV project involved a series of 20 half-day workshops and five follow-up webinars to introduce Go to the Hospital or Stay Here?, the training resources available (12 videos, 5 case studies and Best Practices summary), and strategies for implementation. Altogether 1124 representatives from long-term care facilities, state agencies, ombudsman groups and hospitals attended these interactive workshops. Polled participants reported that resident and family insistence on transfer to the hospital was a major barrier to successful reduction of acute care transfers for them. Those who implemented the Guide facility-wide reported an average 28% reduction in transfers. Primary barriers to facility-wide implementation were lack of leadership, making a choice to absorb the cost of financial penalties and failure to distinguish the goal of Guide use from those of existing transfer reduction programs. The facilities found the workshops helpful preparation for effective implementation of the Guide.

